# Latency to Reward Predicts Social Dominance in Rats: A Causal Role for the Dopaminergic Mesolimbic System

**DOI:** 10.3389/fnbeh.2019.00069

**Published:** 2019-04-05

**Authors:** Laura Lozano-Montes, Simone Astori, Sonia Abad, Isabelle Guillot de Suduiraut, Carmen Sandi, Ioannis Zalachoras

**Affiliations:** Laboratory of Behavioral Genetics, Brain Mind Institute, School of Life Sciences, Ecole Polytechnique Fédérale de Lausanne (EPFL), Lausanne, Switzerland

**Keywords:** social dominance, reward seeking, latency to rewards, ventral tegmental area, optogenetic activations, dopamine and mesolimbic system

## Abstract

Reward signals encoded in the mesolimbic dopaminergic system guide approach/seeking behaviors to all varieties of life-supporting stimuli (rewards). Differences in dopamine (DA) levels have been found between dominant and submissive animals. However, it is still unclear whether these differences arise as a consequence of the rewarding nature of the acquisition of a dominant rank, or whether they preexist and favor dominance by promoting reward-seeking behavior. Given that acquisition of a social rank determines animals’ priority access to resources, we hypothesized that differences in reward-seeking behavior might affect hierarchy establishment and that modulation of the dopaminergic system could affect the outcome of a social competition. We characterized reward-seeking behaviors based on rats’ latency to get a palatable-reward when given temporary access to it. Subsequently, rats exhibiting short (SL) and long (LL) latency to get the rewards cohabitated for more than 2 weeks, in order to establish a stable hierarchy. We found that SL animals exhibited dominant behavior consistently in social competition tests [for palatable-rewards and two water competition tests (WCTs)] after hierarchy was established, indicating that individual latency to rewards predicted dominance. Moreover, because SL animals showed higher mesolimbic levels of DA than LL rats, we tested whether stimulation of mesolimbic DA neurons could affect the outcome of a social competition. Indeed, a combination of optical stimulation of mesolimbic DA neurons during individual training and during a social competition test for palatable rewards resulted in improved performance on this test.

## Introduction

Social inequalities derived from hierarchy establishment have an important impact on individual’s health (Hoebel et al., 2017); in despotic hierarchies most subordinate individuals exhibit a higher prevalence of health problems such as basal hypertension, pathogenic cholesterol profile and increased vulnerability to the atherogenic effects of high-fat diet (Sapolsky, 2005). The establishment of social hierarchies often requires competition between animals to arrange themselves in a priority order (i.e., rank) for the division of resources such as territory, food, water, or sexual partners (Alcock and Rubenstein, 1989; Sapolsky, 2005). Despite the societal and health implications of social status (Sapolsky, 2005; Wilkinson and Pickett, 2006), very little is known about the factors that contribute to the determination of social dominance rank.

Although genetic (van der Kooij and Sandi, 2015) and environmental factors, such as exposure to stressors (Cordero and Sandi, 2007), are known to contribute to the determination of social dominance rank and aggressive behaviors, behavioral dimensions such as individual differences in trait anxiety or motivational processes have been hypothesized to play a key mediating role (van der Kooij and Sandi, 2015). Indeed, recent work has emphasized the involvement of trait anxiety in defining social competitiveness in both rodents (Hollis et al., 2015; Larrieu et al., 2017) and humans (Goette et al., 2015). Moreover, high-dominance individuals have been shown to be faster in decision-making, both in competitive and non-competitive settings, suggesting a general cognitive pattern related to dominance trait (Santamaría-García et al., 2013, 2015; da Cruz et al., 2018).

Laboratory rats form social hierarchies when living in groups, thus they provide an excellent model to study the neuronal mechanisms underlying social behaviors, such as social dominance (Davis et al., 2009; Wang et al., 2014). In the wild, social hierarchies among rodents are established based on displays of offensive behaviors, competitive access to food and water, marking the territory and grooming behaviors among others (Alcock and Rubenstein, 1989; Berdoy et al., 1995). In a laboratory setting, several tests have been designed in order to measure social rank focusing on the natural behaviors that affect hierarchy formation in the wild. Many studies have measured dominance in rats by performing social dominance tests or water and food competition tests. In these tests, animals compete for a new territory (typically through the display of offensive behaviors) or for limited access to water and food, respectively (Peres and Leite, 2002; Cordero and Sandi, 2007; Akers et al., 2008; Timmer and Sandi, 2010; Timmer et al., 2011; Hollis et al., 2015; Larrieu et al., 2017; van der Kooij et al., 2018). Therefore, there is ecological validity for laboratory competition tasks, as in the wild, rats with higher status have more access to natural resources (food, water, among others) while rats with a lower position in the hierarchy often experience reduced access to resources (Berdoy et al., 1995).

Currently, only a few studies have investigated a possible role for individual differences in reward-seeking behavior on dominance (Davis et al., 2009; Balconi and Vanutelli, 2016). For instance, dominant rats on a visible burrow system (VBS) showed higher reward-seeking behavior; however, such behavior was studied after hierarchy establishment, which makes conclusions about causality difficult (Davis et al., 2009). Recent human studies have indicated a possible role of a personality trait, the high Behavioral Activation System (BAS; associated with reward-seeking behaviors), on social competition outcome (Carver and White, 1994; Balconi and Vanutelli, 2016). High-BAS individuals had better performance during an interpersonal competitive task. However, the BAS questionnaires were answered after the individuals had completed the interpersonal competitive phase. This makes it difficult to judge about causality, as participants’ BAS score might have been influenced by the recent performance on the competition task.

Reward-seeking behavior is defined as an activation of the instinctual emotional appetitive state evolved to induce organisms to search and/or approach all varieties of life-supporting stimuli (Alcaro et al., 2007). Reward-seeking is a major modulator of animal behavior; animals will learn to repeat actions that bring them closer to the rewards (Lechner et al., 2000; Hills et al., 2004; Roitman et al., 2004; Wise, 2004). Motivational aspects, including reward-seeking behaviors, have been extensively related to dopamine (DA) projections from the ventral tegmental area (VTA) to the nucleus accumbens (NAc; Alcaro et al., 2007; Nicola, 2007; Arias-Carrión et al., 2010; Luciana et al., 2012; Russo and Nestler, 2013; Ichinose et al., 2017). Reduction of accumbal dopaminergic function has been shown to reduce animals’ exertion of effort to obtain rewards and even to cause failure to respond to reward-predictive cues (Nicola, 2007, 2010; Salamone and Correa, 2012). Furthermore, recent studies have shown that chemogenetic activation of DA neurons in the VTA increased initiation of reward-seeking actions (Boekhoudt et al., 2018). Additionally, optogenetic studies showed a causal role for phasic activation of VTA neurons (mainly projecting to the NAc) on reward-seeking behaviors, social behaviors and motivated behaviors (Adamantidis et al., 2011; Chaudhury et al., 2013; Steinberg et al., 2013; Tye et al., 2013; Gunaydin et al., 2014). These studies have highlighted the role of specific patterns of dopaminergic activity during time-precise behavioral events (such as reward delivery) on the behavioral outcomes (Adamantidis et al., 2011; Steinberg et al., 2013).

In addition, there is indirect evidence from gene expression studies in human (Martinez et al., 2010) and non-human primates (Morgan et al., 2002; Miller-Butterworth et al., 2008; Nader et al., 2012) linking dopaminergic function with social dominance. Dominant monkeys were found to display higher levels of DA metabolites in cerebrospinal fluid samples than subordinate ones (Kaplan et al., 2002). Recent studies from our lab have suggested activation on the dopaminergic projections from the VTA to the NAc and subsequent DA release in the NAc as the underlying neurobiological mechanism by which anxiolytic drugs increase dominance on a social dominance test (van der Kooij et al., 2018).

Here, we examined whether differences in reward-seeking behavior could predict the outcome of a social competition in male rats. We focused on male rats, as they exhibit higher aggression and competitiveness compared to female rats. We hypothesized that individual differences in reward-seeking behavior might predispose high reward-seeking individuals to become dominant when competing for natural resources. We then asked whether these individual differences in reward-seeking behavior are accompanied by differences in the function of the mesolimbic dopaminergic system and whether boosting the dopaminergic output could promote dominance. To this aim, we optically activated DA neurons during a social competition for a limited reward.

Our data showed that reward-seeking behaviors predicted social rank after hierarchy establishment and animals with different reward-seeking behaviors presented different accumbal DA levels. Finally, the outcome of a social competition could be modulated by activating the dopaminergic mesolimbic system in a time-precise manner.

## Materials and Methods

### Animals

**Figure 1:**
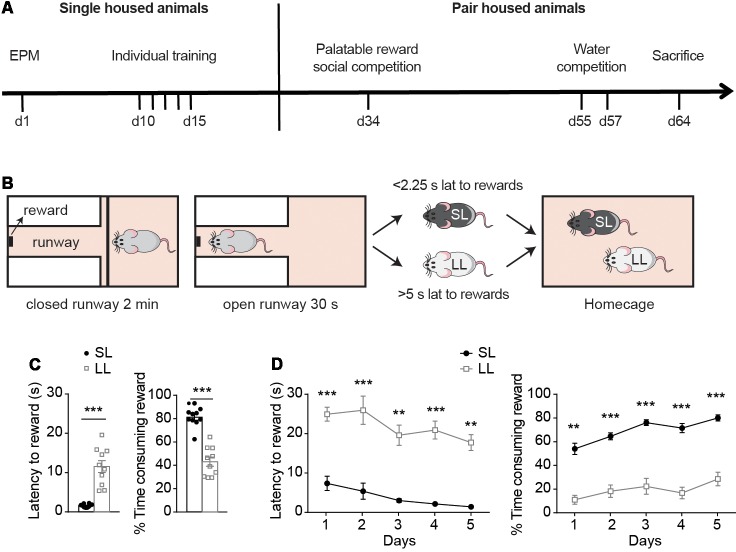
Reward-seeking characterization before hierarchy establishment. **(A)** Experimental timeline, with the battery of tests to which animals were exposed while they were single-housed or pair-housed. **(B)** Criteria used for establishment of LL, SL groups were based on animals’ average latency to rewards during the last day of individual training, when animals had already learned the task. Animals with average latency below 2.25 s or above 5 s, were classified as short latency to reward (SL) and long latency to reward (LL), respectively. **(C)** Behavioral measurements (mean ± SEM) of SL (*n* = 10) and LL (*n* = 10) animals before cohabitation were analyzed. During their last day of individual training, SL rats had lower latency to rewards and spent more time consuming the rewards compared to LL rats. Animals that missed more than one reward on the 5th day of training were re-trained (for two extra days maximum) until they did not miss more than one reward. **(D)** Average latency to rewards and average percentage of time consuming the reward during the first 5 days of individual training of SL and LL animals. **p < 0.01, ***p < 0.001.

Heterozygous transgenic rats expressing Cre recombinase under the control of tyrosine hydroxylase promoter (TH::Cre) on a Long-Evans background were obtained from K. Deisseroth (Witten et al., 2011; McCutcheon et al., 2014). TH::Cre transgenic rats were bred in our animal house at the EPFL by mating Cre-positive founders to wild-type rats; TH::Cre offspring were used in all optogenetic experiments and their wild-type littermates in the rest of experiments. Only male rats aged 12–15 weeks at the initiation of behavioral experiments were used. Animals were housed in a 12 h standard light-dark cycle (lights on from 07:00 to 19:00 h), and food and water were available *ad libitum* (except for water competition task, see below). After weaning, all animals were housed with same-sex littermates. We assumed that this resulted in a very mild dominance relationship; previous studies showed that group housing of littermates had no effects on individual behaviors that were modulated after social defeats (Arakawa, 2006). At the beginning of the experiment, rats were single-housed for a minimum of 7 days, during which handling and habituation phases to the social competition for palatable-reward (SCPR) test (see below) took place. All experiments were carried out in non-food-deprived rats. Thereafter, rats were housed in pairs, with the exception of animals for optogenetics that were single-housed throughout the experiment. Then, social rank established between the two cohabitating rats was determined through several social competition tests as indicated in the timeline scheme (Figure 1A; see also Supplementary Table S1 for an overview of the experiments). All experiments were performed with the approval of the Cantonal Veterinary Authorities (Vaud, Switzerland) and carried out in accordance with the European Communities Council Directive of 22 September 2010 (2010/63/EU).

### Test for Anxiety: Elevated Plus Maze (EPM)

In order to assess trait anxiety, animals underwent the elevated plus maze (EPM) test before starting the individual training for the social competition for palatable rewards (see below; Herrero et al., 2006). This test consists of two opposing open arms (45 × 10 cm) and two opposing closed arms (45 × 10 cm with walls 50 cm high) that extend from a central platform (10 × 10 cm) elevated 65 cm above the floor. Rats were placed on the central platform facing the same closed arm and allowed to explore the maze freely for 5 min. EPM test was performed in the morning, between 9:00 am and 12:00. The behavior of each rat was video recorded and analyzed using a computerized tracking system (Ethovision 3.1.16, Noldus IT, Netherlands). Time spent in the open and closed arms were measured in order to evaluate trait anxiety. Total distance walked in the EPM and the total number of arm entries were measured to assess locomotor activity.

### Open Field Test

In order to assess locomotion differences between SL and LL groups or due to phasic activations of VTA dopaminergic neurons, animals underwent an open field test. The open field consisted of a black circular arena (1 m in diameter, surrounded by walls 32 cm high). For analysis, the total distance walked was calculated. Animals were placed in the center of the arena and their behavior was monitored for 10 min using a video camera that was mounted from the ceiling above the center of the arena. The light was adjusted to 8–10 lx in the center of the arena. Open field test was performed in the morning, between 9:00 am and 12:00. Total distance walked was calculated using Ethovision (Noldus). To test the locomotion of animals receiving optogenetic stimulation, we split the test into two epochs. The first 5 min were without stimulation (light off), while the second epoch (min 5–10) rats received optogenetic stimulation (light on).

### Social Competition for Palatable-Reward (SCPR) Test

The SCPR test was based on a protocol described by Akers et al. (2008) with minor modifications. The apparatus consisted of a testing box made of Plexiglas (52 × 32 × 65 cm). It contained a narrow runway with the reward located at the end of it (Supplementary Figure S1A). The same boxes were used for habituation, individual training and social competition sessions. To habituate rats to the reward, a small amount of melted chocolate (Nutella) was applied with a cotton-tip on the wall of animals’ homecage once a day for three consecutive days. After familiarization with the reward, animals were habituated individually to the box for 3 days. The habituation session consisted of a first phase of 2 min during which animals were confined to one-half of the cage. An opaque divider, that prevented access to the runway, was then removed and rats could explore the whole apparatus for 5 min, accessing the narrow runway and getting the reward. Habituation to both the palatable-reward and the competition box took place always while animals were single-housed.

#### Individual Training Without Competition

Rats were trained individually to enter the runway and consume a small drop of the palatable-reward at the end of the runway. Individual training started around 15:00. On each training day, rats performed six trials. In each trial, following 2 min during which animals were placed in the main area of the box, the divider was removed and animals had 30 s to enter the runway and consume the reward (except for the very first trial in which animals were given 60 s to complete the trial). Rats were trained for a minimum of 5 days. Animals were considered to have learned the task when they missed a maximum of one trial per session. When they missed more than one trial, they received further training sessions until criterion was reached (for a maximum of two extra days). Animals were always single-housed during the individual training period. Only animals that reached the criterion were involved in further experiments. Based on the results from the last day of individual training animals were matched on dyads in order to perform the SCPR test. Wild-type animals were matched for opposite latency to rewards and put to cohabitate together in order to establish a hierarchy. However, in experiments involving TH::Cre animals, they were matched to form pairs with similar latency to rewards and did not cohabitate before the SCPR test. Furthermore, animals in each dyad were matched for age, weight and anxiety levels as measured in the EPM.

#### SCPR Test

Pairs of rats were tested on 12 consecutive trials in a single session. SCPR tests started around 15:00. Rats were marked with either black or blue coloring on the sides of their bodies to distinguish the two rats of each pair. Both rats were introduced to the testing cage simultaneously. Competition testing was conducted in the same way as during individual training.

#### Measurements

The latency to begin reward consumption and the time spent consuming the reward were manually scored by an experimenter blind to the experimental conditions using “Clicker v1.13” software (Toledo-Rodriguez and Sandi, 2007). If a rat did not consume the reward, a latency equal to the maximum trial length (i.e., 30 s) was scored. The latency to begin reward consumption and the time spent consuming the reward were considered for the analysis of the individual training and social competition test (Supplementary Table S2). For classification purposes, the average latency per animal to consume the reward during the six trials of the last training session was considered; animals with average latency below 2.25 s or above 5 s, were classified as short latency to reward (SL) and long latency to reward (LL), respectively. These cut off values were established based on the distribution of the animals’ latencies from the first group we used (Supplementary Figure S1B, left panel). We tried to consider cut-off values that would split the distribution into three subpopulations (LL animals were those falling over the 75th percentile and SL animals were those falling under the 40th percentile), with the intention to study the two extreme subpopulations. This method had the advantage of clearly separating LL rats and splitting the rest (which fell in a shorter range of latencies) almost in two groups containing equal numbers of rats. The distribution of all animals used in the study was, based on our cut-off values, approximately 27% for LL rats, 36% for intermediate latencies (>2.25 and <5 s) and 37% for SL (Supplementary Figure S1B, right panel), suggesting that groups were fairly consistent between different cohorts.

### Water Competition Test (WCT)

Same pairs of cohabitating rats that performed the SCPR test (described above) underwent water competition tests (WCTs) 23 days after SCPR test completion. The pairs of rats were deprived of water mainly during the dark cycle from 00:00 to 08:00. Then, a 5-min WCT was performed by placing a bottle of water in the feeder holder of their homecage during the light cycle in the housing room. WCT tests were manually scored by an experimenter blind to the conditions with the aid of Clicker v1.13 software (Toledo-Rodriguez and Sandi, 2007). Latency to first drink and the duration of drinking behavior for each rat within each dyad were measured. The animal in the dyad that drank more was considered the dominant rat, as previously reported (Baenninger, 1970; Lucion and Vogel, 1994; Cordero and Sandi, 2007).

### High Performance Liquid Chromatography (HPLC) Analysis of Monoamine Level in Brain Samples

Animals were decapitated and their brains were quickly removed, frozen in isopentane on dry ice, at a temperature between −50 and −40°C, and stored at −80°C until further processing. Coronal sections (200-μm thick) were punched to obtain the brain tissue of NAc region as previously described (Guitart et al., 2000). Brain samples were briefly sonicated in Eppendorf vials containing 120 μl of 0.1 M perchloric acid (PCA) and centrifuged at 16,000 *g* for 10 min at 4°C. The supernatant was collected and used for high performance liquid chromatography (HPLC) analysis. Two samples of SL rats were lost during the extraction process. Levels of DA, as well as DA metabolites 3,4-dihydroxyphenylacetic acid (DOPAC) and homovanillic acid (HVA), were assessed by reverse-phase HPLC with electrochemical detection (HPLC-ECD stand-alone system, HTEC-500). Using a mobile phase, consisting of 20% methanol 8.85 g/l citric acid monohydrate, 200 mg/l octane-1-sulfonic acid sodium salt, 5 mg/l EDTA, 3.11 g/l sodium acetate dissolved in Milli-Q water, the different catecholamines were separated in a reversed phase separation column EICOMPACK SC-3ODS.

### Virus Injection and Implantation of Optical Fibers

Standard stereotaxic procedures were used to infuse the virus, as previously described (Witten et al., 2011). TH::Cre transgenic rats received unilateral VTA injections of AAV5 (1.5 × 10^12^ particles/ml) with the following constructs from the University of North Carolina Vector Core: EF1a-DIO-hChR2(H134R)-EYFP for cre-inducible expression of channelrhodopsin 2 (ChR2) or EF1a-DIO- EYFP as control.

Two small burr holes were drilled unilaterally over the VTA at the following coordinates: AP −5.3 and −6.3; ML ± 0.7 (the hemisphere of injection was randomly selected), as previously described (Tye et al., 2013). A custom-made 26 gauge infuser was used to deliver 1.0 μl of virus at two depths in each hole (DV −8.2 and −7.0, all coordinates from skull surface) for a total of 4.0 μl virus delivered unilaterally to the VTA. Each 1.0 μl of virus was infused at a speed of 0.1 μl per minute using a syringe pump (Harvard Apparatus). The virus infuser was left in place for an additional 10 min following each injection before it was slowly removed.

Four weeks after viral infusion was performed, animals underwent a second surgery to insert the optic fibers into the VTA (AP −5.8; ML ± 0.7; DV −7.5), as previously described (Tye et al., 2013). The implanted optic fiber was 240 μm outer diameter (Doric lenses) and was secured to the skull surface with five stainless steel screws and dental cement. All behavioral tests were conducted >5 weeks post- viral injection surgery.

### Optical Activations

A 200-μm patch cord was connected to the external portion of the chronically implantable optical fiber with a zirconia sleeve (Doric Lenses). Patch cords were attached through a rotatory joint (Doric lenses) to a 473-nm blue laser diode (Dreamlasers), and light pulses were generated through a stimulator (Agilent technologies). Optical-fiber light power from the patch cord was measured using a light sensor (Thorlabs) before each animal was connected to a patch cord, in order to check that laser intensity was constant among animals. Light intensity ranged from 1.5 to 2 mW at the end of the patch cord for each session. For effects of phasic stimulation of DA neurons during individual training, both ChR2-expressing rats and control animals were optically stimulated during the 30 s of reward availability by 20 pulses at 20 Hz, 5 ms pulse duration every 5 s. During SCPR experiments the phasic protocol of stimulation lasted the whole test and was 20 Hz, 20 pulses, 5 ms pulse duration every 5 s.

### Intracranial Self-Stimulation (ICSS) Test

In order to validate our optogenetic activation protocol, we aimed to induce intracranial self-stimulation (ICSS) by phasically activating DA neurons in the VTA. This behavioral effect in response to optogenetic activations has been previously reported (Witten et al., 2011). Experimental sessions were conducted in operant conditioning chambers (25.4 × 30.5 × 25.4 cm^3^; Coulbourn Instruments, Bilaney Consultants) contained within sound-attenuating cubicles. The left panel was fitted with two nosepoke ports, each with three LED lights at the rear. Prior to training sessions, rats were gently attached to patchcord cables for optical stimulation. Optical stimulation was controlled by a computer running Graphic State 2 (Coulbourn instruments) software, which also recorded responses at both nosepoke ports. The protocol followed was the same as previously described by Witten et al. (2011). For all test sessions, the start of a session was indicated to the rat by the illumination of a white house light. During the first day of training, both active and inactive nosepoke ports were baited with a chocolate cereal treat to facilitate the initial investigation. Rats were given four daily sessions of 2 h each in which they could respond freely at either nosepoke port. For all rats (ChR2 and controls), a response at the active port resulted in the delivery of a 1 s train of light pulses (20 Hz, 20 pulses, 5 ms duration). Concurrently, the LED lights in the recess of the active port were illuminated, providing a visible cue whenever stimulation was delivered. Responses at the active port made during the 1 s period when the light train was being delivered were recorded but had no consequence. Responses at the inactive port were always without consequence.

### Histology

Specificity of ChR2-EYFP expression in DA neurons and viral injections and optic fiber placements were verified *post hoc*. Animals were decapitated, brains were rapidly isolated and transferred to a 4% Paraformaldehyde (PFA) solution at 4°C for 24 h. Subsequently, the brains were transferred to a 30% sucrose solution at 4°C for 48 h. Then brains were frozen in isopentane on dry ice and finally stored at −20°C. Immunohistochemical detection of eYFP and tyrosine hydroxylase was performed similarly as previously described (Witten et al., 2011). Free-floating coronal sections of 40 μm were washed with TBS 1× 0.3% triton. Subsequently, brain sections were blocked with 20% donkey serum for 2 h. Goat polyclonal anti TH antibody (1:1,000 dilution, ab101853 from Abcam) was incubated overnight at 4°C. Sections were then washed with TBS 1× 0.3% triton and incubated with (1:600 Alexa 568 donkey antigoat) for 2 h. Finally, sections were mounted onto microscope slides in phosphate-buffered water and coverslipped with Vectashield mounting medium that contained a DAPI nuclear counterstain (Reactolab SA). ChR2-YFP expression and fiber optic placement into the VTA were checked in all rats used for behavioral experiments. No animal needed to be removed due to lack of viral expression or optic fiber misplacement within the VTA.

### Statistical Analyses

Sample sizes (*n*) are indicated in figure legends and represent biological replicates. Sample sizes were calculated based on pilot experiments and animals were allocated to different groups based on characterization criteria (short latency to rewards and low latency to rewards) or randomly allocated to a group (ChR2 or control). Behavioral scoring and experimental assessment were performed by experimenters blind to treatment groups. Unpaired two-tailed Student’s *t*-tests were used to compare sets of data obtained from independent groups of animals. Welch correction was used for the analysis of the latency to reward during the last day of individual training, as the samples did not have equal standard deviations. Paired two-tailed Student’s *t*-tests were used to compare behavioral data within dyads. In the data sets from the WCTs, the percentage of drinking was compared using one-sample *t*-test against the level of chance (50%). Two-way ANOVA was used in order to analyze the individual data on the training sessions between days in both SL/LL and ChR2/Control groups. Two-tailed Mann-Whitney test with Bonferroni correction was used in order to analyze the responses to the active and inactive ports during the ICSS test. All data were analyzed using Prism version 5.01 (Graphpad Software, San Diego, CA, USA). Data are presented as the mean ± SEM and statistical significance is considered at *p* < 0.05.

## Results

### Individual Differences in Latency to Approach a Reward Predict Social Rank

Single-housed, rats were exposed to five training days (at least) and classified according to their average latency to obtain a palatable-reward during the last day of individual training, as animals with either short latency (SL; latency below 2.25 s) or long latency to reward (LL; latency above 5 s; Figure 1B). During the last day of individual training, SL animals had significantly lower latencies to rewards than LL animals (*t* = 6.477, *df* = 9, *p* < 0.0001; Figure 1C) and spent more time consuming the rewards (*t* = 8.069, *df* = 18; *p* < 0.0001; Figure 1C). As indicated in “Materials and Methods” section, the study only involved animals that readily consumed the food reward in their homecage (Supplementary Figure S1C) and learned the task during individual training. Training criterion was to miss a maximum of one trial per session. When animals missed more than one trial, they received further training sessions until the criterion was reached (Supplementary Figure S1D, for a maximum of two extra days). Animals classified as either SL or LL on the basis of their latencies on the last training day, showed as well different latencies to the reward and in the percentage of time spent consuming the reward across the different training days. Two way ANOVA followed by Bonferroni post-tests indicated differences between SL and LL animals on their latency to rewards (Group effect: *F*_(1,18)_ = 113.2, *p* < 0.0001, *t* = 4.421, *p* < 0.01; *t* = 4.756, *p* < 0.001; *t* = 5.526, *p* < 0.001; *t* = 5.632, *p* < 0.001; *t* = 5.292, *p* < 0.001, for days 1–5 respectively); and in the time spent consuming rewards (Group effect *F*_(1,18)_ = 131.3, *p* < 0.0001, *t* = 4.421, *p* < 0.01; *t* = 4.756, *p* < 0.001; *t* = 5.526, *p* < 0.001; *t* = 5.632, *p* < 0.001; *t* = 5.292, *p* < 0.001, Figure 1D) on each of the first 5 days of training. These differences were also present when comparing the last 5 days of individual training of each animal (Supplementary Figure S1E, *p* < 0.001, *t* = 5.2305, *p* < 0.001, *t* = 6.9468, *p* < 0.001, *t* = 6.9504, *p* < 0.001, *t* = 6.6204, *p* = 0.0034, *t* = 3.5221, for latency the last 5 days of training (1–5, respectively) and, *p* < 0.001, *t* = 6.4513, *p* < 0.001, *t* = 6.9865, *p* < 0.001, *t* = 10.075, *p* < 0.001, *t* = 8.8382, *p* < 0.001, *t* = 6.0973 for % time consuming rewards, in the last five of individual training (1–5, respectively).

Following this behavioral characterization, pairs constituted of one SL and one LL rat were matched for similar body weight and anxiety levels (Supplementary Figure S1F; both traits have been shown to affect social dominance; Barnet, 1958; Hollis et al., 2015). Behavioral measurements from the EPM and open field tests were analyzed, confirming that there were no differences in anxiety levels (*p* > 0.05, *t* = 0.934 for % time in open arms, *p* > 0.05, *t* = 0.732 for latency to open arms and *p* > 0.05, *t* = 0.440 for total number of arm entries), or total locomotion between SL and LL animals (*p* > 0.05, *t* = 0.523 for distance moved in the EPM and *p* > 0.05, *t* = 1.309 for distance moved in the open field) that could represent an advantage for the social competition tests (Supplementary Figures S1F,G). Pairs of SL and LL animals were then left to cohabitate for 17 days.

**Figure 2:**
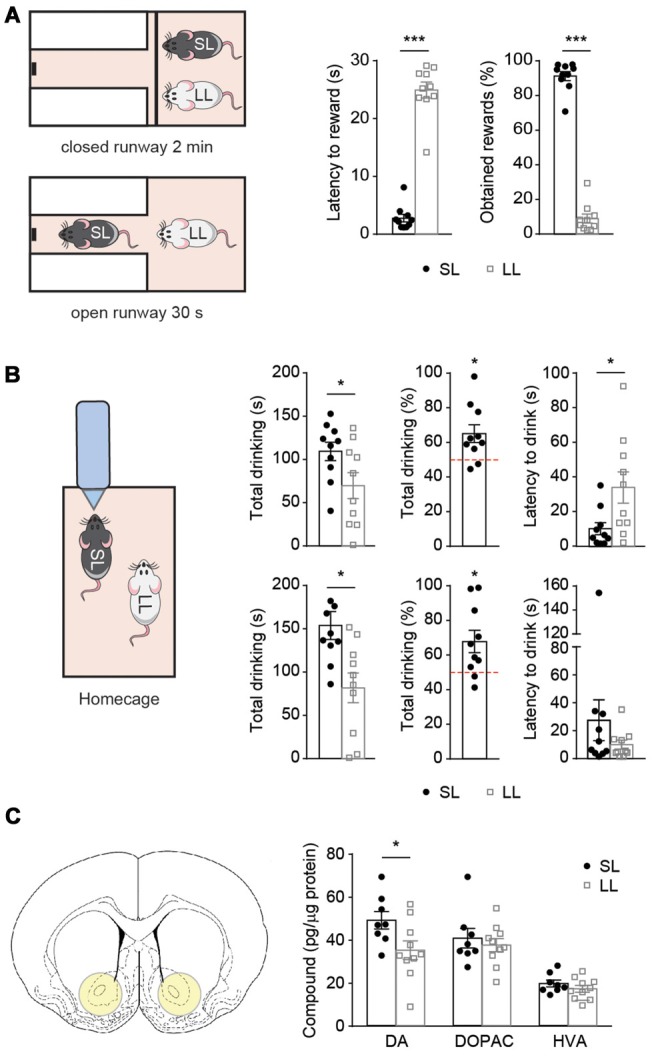
Latency to reward predicts the outcome of several social competition tests after hierarchy establishment. Short and long latency animals show differences on accumbal dopamine (DA) levels. **(A)** Pairs of SL-LL animals (*n* = 10) performed the social competition for palatable-reward (SCPR) test after cohabitation. SL rats had lower latency to rewards and spent more time consuming the rewards compared to LL rats. **(B)** Same pairs of SL-LL animals performed two water competition tests (WCTs). SL rats drank more than LL rats and their water consumption was significantly higher than chance level. They also had lower latency to the initial drink compared to LL rats. In a second WCT, SL rats drank more, also compared to chance level, but without having differences in latency to initial drink (mean ± SEM). **(C)** Schematic of accumbal tissue collection for neurotransmitter analysis. Accumbal levels of DA, 3,4-dihydroxyphenylacetic acid (DOPAC) and homovanillic acid (HVA; mean ± SEM) in SL (*n* = 8) and LL (*n* = 10) animals used for previous social competition experiments were analyzed here. SL rats had higher DA content in the nucleus accumbens (NAc) compared to LL rats. No difference was observed in DOPAC or HVA content between the groups. *p < 0.05, ***p < 0.001.

Subsequently, we evaluated if cohabitating SL and LL animals would differ in their ability to reach the palatable reward when competing for its access (Figure 2). SL animals retained lower latencies to reward (*t* = 11.74, *df* = 9, *p* = 0.0001; Figure 2A) and obtained a higher percentage of rewards (*t* = 15.83, *df* = 9, *p* < 0.0001; Figure 2A, *p* = 0.0020; Figure 2A) during the competition test than their LL counterparts.

In order to assess whether the advantage of SL rats to win a competition could be extended to competitions involving rewards beyond the one used for the group stratification and a test where the fastest animal is not necessarily the dominant one, the same pairs of animals were subsequently submitted to two WCTs. Before each test, the pair of cohabitating animals was water-deprived overnight. Then, they were given access to a single water bottle for 5 min, the bottle access was not restricted, therefore the slower animal could also approach the bottle and push away the animal that was drinking (Figure 2B). In both tests, SL animals displayed an advantage over LL ones in accessing water, displaying lower latencies to drink (*t* = 2.547, *df* = 9, *p* = 0.0314; Figure 2B) and spending more time drinking than their LL counterparts (*t* = 3.149, *df* = 9, *p* = 0.0118; Figure 2B). We also verified that the percentage of drinking by SL rats was different from chance level (50%; one sample *t*-test *t* = 2.928, *df* = 9, *p* = 0.0168; Figure 2B). Similar results were obtained on a subsequent WCT performed 2 days afterwards; in terms of time drinking (*t* = 2.551, *df* = 9, *p* = 0.0311; Figure 2B; test against chance level (*t* = 2.742, *df* = 9, *p* = 0.0228; Figure 2B), except for the latency to drink that was not significantly different (*t* = 1.101, *df* = 9, *p* = 0.2995; Figure 2B).

These data suggest that reward-seeking behavior plays an important role in hierarchy establishment, as indicated by the outcome of social competition tests that involved different sensory-motor functions. Importantly, the results of the second WCT, where LL animals did not differ in the latency to drink, but they still spent less time drinking than the SL rats, indicate that the establishment of social hierarchy did not merely depend on how fast animals could access the resource, but that SL animals could use other strategies to become dominant, such as pushing the LL animals away in order to ensure prolonged access to the resource.

### Individual Differences in Latency to Reward Are Accompanied by Differences in Accumbal Dopamine Levels

Given that DA has been implicated in the locomotor approach to reward-associated cues (Nicola, 2010), we compared levels of accumbal DA and its metabolites between SL and LL animals. NAc tissue punches were taken at basal conditions 1 week after the last WCT. We found higher DA levels in SL than in LL rats (*t* = 2.296, *df* = 16, *p* = 0.0355; Figure 2C), but no differences in the levels of DOPAC and HVA (*t* = 0.6110, *df* = 16, *p* = 0.5498; *t* = 1.100, *df* = 16, *p* = 0.2877, for DOPAC and HVA, respectively; Figure 2C). As we could only measure DA levels in the NAc post-mortem, we aimed to subsequently study the causal effects of DA modulation on the outcome of a SCPR test.

### Optogenetic Activation of Mesolimbic Dopamine Neurons Increases Social Dominance

Phasic optogenetic activation of VTA neurons mainly projecting to the NAc has been causally linked to reward-seeking behaviors (Adamantidis et al., 2011; Steinberg et al., 2013). Furthermore, in the same transgenic TH::Cre rat model as used here, phasic activation (20 Hz) of VTA DA neurons was shown to increase DA levels in the NAc and induced ICSS (Witten et al., 2011). Here, TH::Cre rats were infused with a Cre-inducible AAV expressing ChR2-eYFP into the VTA (Supplementary Figure S2A). Control animals were TH::Cre rats that received an infusion of Cre-inducible AAV expressing only eYFP into the VTA. Histological analyses confirmed that ChR2-eYFP expression was restricted to the VTA (Supplementary Figure S2A).

We first aimed to verify the feasibility of our optogenetic approach by replicating the optogenetic induction of self-stimulation in an operant task (Witten et al., 2011). Rats were given the opportunity to freely respond at two identical ports (Supplementary Figure S2B). Nosepokes at the active port induced phasic activations of VTA DA neurons, whereas nosepokes at the inactive port had no consequence. ChR2 expressing rats made significantly more responses in the active port than control animals did during each one of the 4 days of training (*U* = 0, *p* = 0.0079; *U* = 0, *p* = 0.0119; *U* = 0, *p* = 0.0079; *U* = 0, *p* = 0.0357; Supplementary Figure S2C). This indicates that phasic dopaminergic stimulation was correctly delivered in our experiments as it induces vigorous ICSS, as previously reported (Witten et al., 2011).

**Figure 3:**
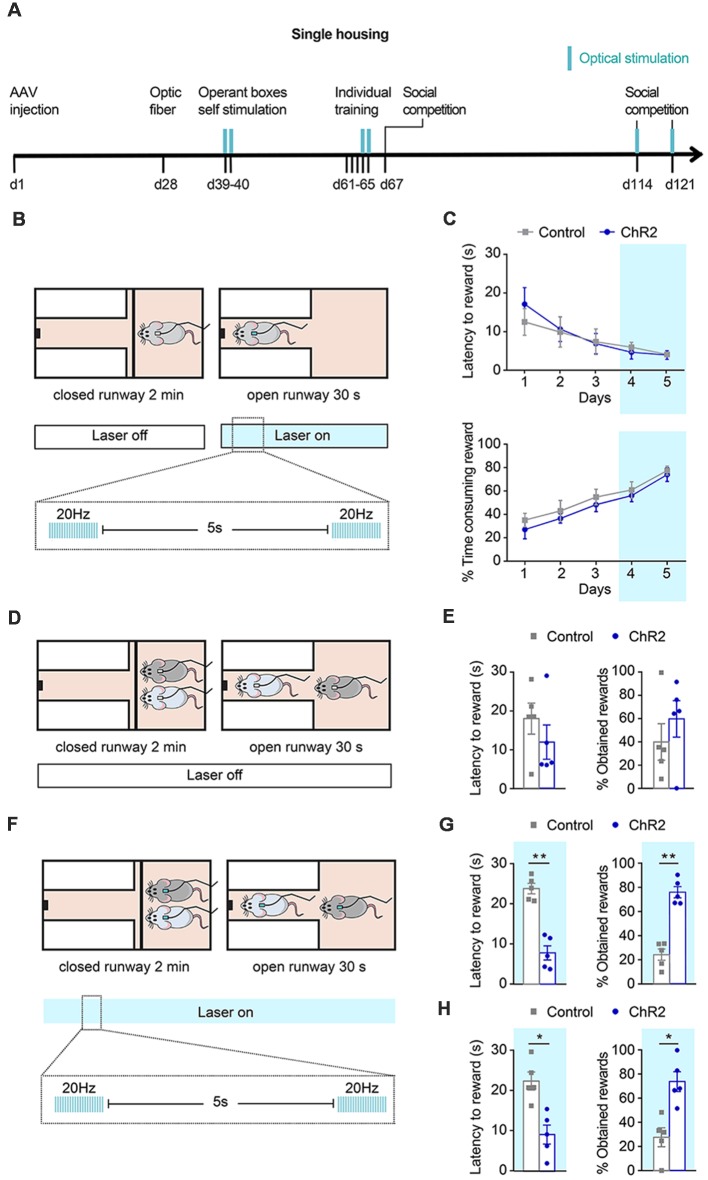
Effects of phasic DA during individual training and social competition in dominance. **(A)** Experimental timeline followed by a group of channelrhodopsin 2 (ChR2; *n* = 5) and control (*n* = 5) animals to study the effects of phasic DA activations during individual training and competition on the SCPR test outcomes. Blue marks indicate sessions in which optical stimulation was applied. **(B)** Schematic representation of illumination pattern used during the last 2 days of the individual training in a group of ChR2 (*n* = 5) and control (*n* = 5) animals. **(C)** Average latency to rewards and percentage of time consuming the reward (mean ± SEM) during each day of individual training for ChR2 and control animals. **(D)** Animals underwent a social competition test without optical activation. **(E)** No difference in average latency to rewards and percentage of obtained rewards (mean ± SEM) for ChR2 and control animals during a social competition test. **(F)** Animals underwent two further social competition tests while their ventral tegmental area (VTA) DA neurons were phasically activated. **(G)** Average latency to rewards was lower in ChR2 rats and percentage of obtained rewards was higher compared to control rats (mean ± SEM) for the same pairs of ChR2 and control animals. **(H)** Average latency to rewards and percentage of obtained rewards (mean ± SEM) from new pairs of ChR2 and control animals (*n* = 5), which had never encountered each other before, during a social competition test. Average latency to rewards was lower in ChR2 rats and percentage of obtained rewards was higher compared to control rats (mean ± SEM). Blue background on the graphs indicates sessions during which optical activation was used. *p < 0.05, **p < 0.01.

Then, we aimed to study if phasic activation of VTA DA neurons during reward acquisition would be sufficient to change the outcome of a SCPR test. We first assessed whether optogenetic activation of DA neurons in the VTA during individual training in one of the two subsequently competing animals would affect the subsequent outcome of the dyadic competition (Figure 3A). Animals performed 3 days of training without any stimulation. Then, during the last 2 days of training, animals were given phasic optogenetic stimulation while they accessed the rewards (Figure 3B). Optogenetic stimulation did not result in differences in performance during training. Two way ANOVA followed by Bonferroni post-tests indicated no differences between control and ChR2 animals on their latency to rewards (Group effect: *F*_(1,39)_ = 0.141, *p* = 0.71, *t* = 1.148, *p* > 0.99; *t* = 0.184, *p* > 0.99; *t* = 0.118, *p* > 0.99; *t* = 0.309, *p* > 0.99; *t* = 0.037, *p* > 0.99, for days 1–5 respectively); or in the time spent consuming rewards (Group effect *F*_(1,39)_ = 2.122, *p* = 0.153, *t* = 0.906, *p* > 0.99; *t* = 0.721, *p* > 0.99; *t* = 0.734, *p* > 0.99; *t* = 0.511, *p* > 0.99; *t* = 0.395, *p* > 0.99) on any training day (Figure 3C). We found no effect of the treatment when pairs of formerly stimulated ChR2 and control rats were allowed to compete for the palatable-reward (Figure 3D), as indicated by equivalent latency to reward (*t* = 0.7325, *df* = 4, *p* = 0.5045; Figure 3E) and percentage of obtained rewards (*t* = 0.6319, *df* = 4, *p* = 0.5618; Figure 3E). Subsequently, in the same pair of rats that had undergone optogenetic activation of DA neurons during the last 2 days of individual training, we performed a second SCPR test in which animals received phasic DA activations (Figure 3F). We found lower latencies to reward (*t* = 5.384, *df* = 4, *p* = 0.0058; Figure 3G) and a higher percentage of obtained rewards (*t* = 5.522, *df* = 4, *p* = 0.0053; Figure 3G) in ChR2 than in control animals.

Given that the same pair of animals had previously competed against each other, a carry-over effect in the last part of the experiment could not be ruled out. Therefore, we performed a further experiment in which new pairs of animals from the same cohort were matched based on their similar latency to rewards during the last training day and were put together for the first time for an SCPR test while DA neurons of the ChR2 rats were phasically activated and control rats received only the same light pulses into their VTA. As hypothesized, ChR2 rats displayed lower latency to reward (*t* = 2.911, *df* = 4, *p* = 0.0436; Figure 3H) and obtained a higher percentage of rewards (*t* = 2.884, *df* = 4, *p* = 0.0448) than controls (Figure 3H).

**Figure 4:**
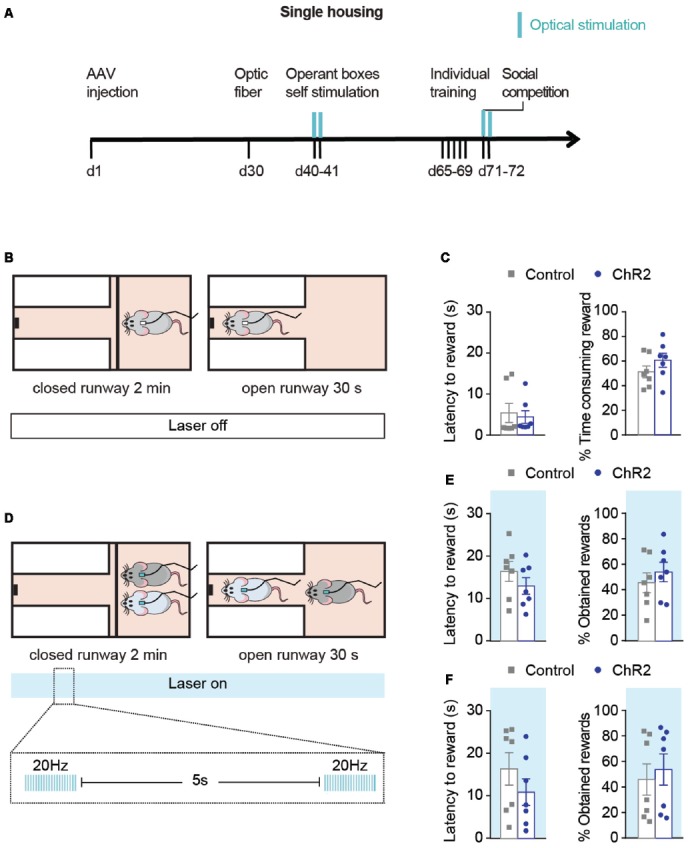
Optical activation during social competition test only does not modulate the outcome of the competition. **(A)** Experimental timeline followed by a group of ChR2 (*n* = 7) and control (*n* = 7) animals to study the effects of phasic DA activations during competition on the SCPR test outcomes. Blue marks indicate sessions in which optical stimulation was applied. **(B)** Rats underwent individual training without optical activations. **(C)** Latency to rewards and percentage of obtained rewards (mean ± SEM) on day 5 of individual training for ChR2 (*n* = 7) and controls (*n* = 7). **(D)** During the SCPR test, animals underwent optical activations of VTA dopaminergic neurons. **(E)** Latency to rewards and percentage of obtained rewards (mean ± SEM) on the same animals during SCPR test while phasic activations of dopaminergic neurons in the VTA. No differences were observed between the groups. **(F)** Latency to rewards and percentage of obtained rewards (mean ± SEM) on the same animals during a second SCPR test while phasic activations of dopaminergic neurons in the VTA. No differences were observed between the two groups.

In order to study whether DA activations during competition were sufficient to change the outputs of the SCPR test, we performed an additional experiment in a different group of animals in which activation of DA neurons took place only during the SCPR test was not affected (Figure 4A). Animals underwent individual training for the SCPR test without DA activations (Figure 4B). There were no differences between control and ChR2 animals’ performance on their last day of individual training. Control and ChR2 animals did not differ on their latency to rewards (*t* = 0.3418, *df* = 12, *p* = 0.7384; Figure 4C) or in the time they spent consuming the rewards (*t* = 1.250, *df* = 12, *p* = 0.2352). Afterward, pairs of ChR2 and control animals underwent optical activations of the VTA during the SCPR test, as previously described, in order to study if DA activations during the competition were enough to modulate the outputs of the SCPR test (Figure 4D). We found no effect of the treatment when pairs of ChR2 and control rats were allowed to compete for the palatable-reward (Figure 4E), as indicated by equivalent latency to reward (*t* = 0.5522, *df* = 6, *p* = 0.6007; Figure 4E) and percentage of obtained rewards (*t* = 0.8439, *df* = 6, *p* = 0.4311; Figure 4E). Subsequently, in the same pair of rats that had undergone optogenetic activation of DA neurons during the SCPR test, we performed a second SCPR test in which animals also received phasic DA activations, in order to investigate whether several days of DA activations were required to have the effects on SCPR outputs (Figure 4F). We found no effect of the treatment when pairs of ChR2 and control rats were allowed to compete for the palatable-reward (Figure 4E), as indicated by equivalent latency to reward (*t* = 0.3251, *df* = 6, *p* = 0.7561; Figure 4F) and percentage of obtained rewards (*t* = 0.8154, *df* = 6, *p* = 0.4460; Figure 4F). Therefore, our results indicated that a combination of phasic stimulation during both training and the SCPR test were required in order to enhance animals’ competitiveness in this test.

## Discussion

Our results are in line with previous studies showing that dominant rats present higher reward-seeking behaviors (Davis et al., 2009) and recent human studies indicating that high reward-seeking improves performance of a competitive task (Balconi and Vanutelli, 2016). However, these studies measured reward seeking behaviors after hierarchy has already been established, making difficult to conclude if differences in reward-seeking behaviors influence the social rank acquisition or if the high social rank acquisition produces changes in reward-seeking behaviors. Here, we show that differences in reward-seeking behaviors prior to hierarchy establishment can predict the outcome of several social competition tests after hierarchy has been established. Hence, we provide evidence in support of reward-seeking behavior functioning as a personality trait in rats related to dominance and the formation of social hierarchies. Our data suggest that this trait is related to accumbal DA content, thus it may be important to use latency to approach a reward for the characterization of individual differences in outbred rat populations, together with assessments of anxiety or exploratory behavior.

Animals were classified based on their latency to get a palatable-reward when given temporary access to it, individually. The selection of latencies to reward as a measurement of reward-seeking behavior is supported by human studies showing that high reward-seeking individuals have lower latencies to react to pleasant pictures (Aluja et al., 2015). Furthermore, deficits in reward-seeking behavior of animals treated with DA receptor antagonists were due to an increased latency to initiate approach behavior towards the reward (Nicola, 2010). Conversely, high dominance trait in humans is accompanied by faster decision making (da Cruz et al., 2018). As our animals were classified based on the latency to acquire rewards, we additionally used a different kind of social competition test in which the latency to arrive to the reward does not play a role in dominance. This is the case of the WCT, in which the access to the bottle is not limited and therefore the animal arriving second can still push the other one away and drink. On the second WCT we did not see any differences between SL and LL animals in the latencies to drink, however, the SL animals pushed the LL away and still drank more.

In addition, our results showed increased levels of accumbal DA in the SL animals, as measured after the hierarchy was established. Consistently, a great number of studies have indicated a role for the dopaminergic reward system in dominance (Grant et al., 1998; Overli et al., 1999; Kaplan et al., 2002; Morgan et al., 2002; Martinez et al., 2010; Hollis et al., 2015). However, most of these studies (including ours) measured DA levels after hierarchy establishment making difficult any assumption on its causality on dominance. However, individual differences in dopaminergic responses to a new situation (presence of a competitor) during a social competition might determine the chances of becoming dominant. Accordingly, previous studies from our lab have shown that activation of accumbal neurons expressing the D1 receptor during social competition was highly correlated with animals’ performance (highlighting that activation of DA neurons during competition might play a critical role; van der Kooij et al., 2018).

It is important to mention that even though we found differences in accumbal DA levels, we did not find differences in DA metabolite levels (DOPAC and HVA), which could suggest lack of differences in DA signaling or turnover. Since our measurement of DA and its metabolites is limited only to analysis under basal conditions, without multiple time points, we cannot make any inferences regarding DA signaling/turnover. However, other explanations may also exist: first, we measured total neurotransmitter levels, not solely extracellular levels, therefore, it is not possible to draw any conclusions regarding levels of DA release. Thus, even though SL rats had higher DA levels compared to LL rats, this may represent DA that can be released upon presentation of a certain stimulus (i.e., social competition or involvement in a task for the acquisition of a reward), rather than constitutive high levels of DA release. Secondly, DOPAC and HVA levels do not depend solely on DA levels, but rather to a combination of factors including DA synthesis rate, levels of DA release and utilization and expression levels of the metabolizing enzymes (Monoamine oxidase for DOPAC and Monoamine oxidase and Catechol-O-methyltransferase for HVA). Therefore, an 1-1 relationship between DA and metabolite levels cannot always be found (Sharp et al., 1986; Soares-Da-Silva and Garrett, 1990).

In accordance with our data, previous studies using rats selected for different traits have often found relations between the activity of the dopaminergic system and individual differences in behaviors related to reward-seeking or motivation. For example, work done in rats selectively bred for their locomotor response to novelty has shown that “high-responders” (individuals displaying high locomotor response to novelty) also display higher sensation-seeking and risk-taking behaviors, while they also display a hyperdopaminergic state, compared to “low responders” (individuals displaying low locomotor response to novelty; Flagel et al., 2014). Similarly, Roman high- (RHA) and low-avoidance rats (RLA) display differences in their dopaminergic signaling. In this model of rats selectively bred for rapid or poor acquisition of active avoidance (Driscoll et al., 1998), RHA rats display higher preference and intake of palatable food and higher DA release in response to cocaine, compared to RLA rats (Giorgi et al., 2007). These findings, including ours, suggest that there may be a direct association between increased dopaminergic tone and reward-seeking behaviors oriented towards drug consumption, palatable food intake or even sexual behavior (Melis et al., 2018). Given that reward-seeking behaviors (strongly associated with DA) predicted hierarchy establishment, in our experiments, we hypothesized that mesolimbic dopaminergic responses (during training and/or competition session) might have a causal role on the SCPR outcome.

Recently it has been proven that phasic DA stimulation of the VTA unilaterally delivered on a time-precise manner (during reward delivery) was sufficient to improve the learning of the reward acquisition paradigm (Steinberg et al., 2013). Similar phasic DA activations of the VTA have been shown to modulate reward seeking, motivation and social behaviors, closely related to dominance (Adamantidis et al., 2011; Tye et al., 2013; Gunaydin et al., 2014). Furthermore, synaptic DA release in the NAc depends on neuronal activity in the VTA (Sombers et al., 2009) and phasic unilateral activation of VTA DA neurons has been shown to increase DA levels on the NAc by using voltammetry *in vivo* (Witten et al., 2011) and even lead to sustained DA release for several minutes, as measured by microdialysis (Lohani et al., 2018). It is important to note that in our study we used unilateral stimulation of VTA neurons, which was shown to be effective in previous studies.

Previous studies have highlighted the role of individual differences in DA responses in reversal learning and flexible approaches in relation to reward acquisition (Nicola, 2010; Klanker et al., 2015; Boekhoudt et al., 2018). Moreover, individual differences in phasic DA release evoked by the new rewards predicted reversal learning behavior (Klanker et al., 2015). Individuals that have an elevated DA response associated with the reward acquisition have also higher chances to develop a flexible approach (such as reverse learning) in order to improve the chances of reward acquisition in a new situation in which a different behavior is required in order to get the reward. Therefore, we studied whether an enhancement of the endogenous DA release during the acquisition of the palatable rewards on the last 2 days of individual training could also increase the chances of the animals to get more rewards during the SCPR test, perhaps by developing a flexible strategy (such as pushing the other animal away).

Our phasic activations were time-locked to the reward acquisition epochs (30 s where the animal could access the palatable reward) in order to enhance their endogenous levels of DA to the reward acquisition. We did not observe any changes on their average latencies to rewards or the time-consuming rewards during the two last sessions where DA activations occurred. This could have been because animal latencies were already very low and it was not possible to decrease further. Furthermore, previous studies have shown that DA is required for reward-seeking behaviors only when the specific actions to obtain the reward vary (when different actions are required to reach the reward), or when animals need to develop a new behavioral strategy to acquire the reward (van der Meer and Redish, 2009; Nicola, 2010), which was not the case for the last two training sessions.

Our findings indicated that the phasic DA activations during the last 2 days of training were not sufficient to fully shift the outcome of the SCPR in favor of the ChR2 rat in all competing pairs. Nevertheless, in the majority of pairs, the ChR2 rat won the competition even in the SCPR test without phasic DA activations. Furthermore, when adding a previous cohort of animals that received optical stimulation during the last 2 days of individual training and performed SCPR test without optical activations, we also observed a similar pattern of only a few animals that did not win the SCPR test (Supplementary Figure S3). It is worth noting that in the ChR2 rats that did not win the competition following phasic DA activations only during training, we could not identify any issues with viral delivery/expression, optic fiber implantation or any other issue that would result to exclusion of these rats.

In line with a previous study indicating that the accumbal neurons that fired during reward delivery also fired when animals decided which actions to take during flexible (but not inflexible) approach (van der Meer and Redish, 2009), our findings indicated that a combination of phasic DA activations during the last 2 days of individual training and during SCPR test were sufficient to enhance dominance (Figure 3G) and shift the outcome of the competition in favor of the ChR2 rat in all pairs. Importantly, when we performed a new SCPR test with new pairs, we found the same effect of optical activation, suggesting that the result could not be attributed to a carry-over effect of the first SCPR test done with the same pairs.

In contrast, phasic activations of DA neurons only during the SCPR test were not enough to shift dominance in favor of the ChR2 group in the SCPR test (Figure 4E), highlighting the critical effect of the phasic DA activations during training. The lack of effect of the phasic DA activations during a second SCPR test suggests that the effect of the combination of phasic DA activations during individual training and during the SCPR test is not merely due to the repeated activation of DA neurons, but that the timing and context of the activations is crucial. Finally, the comparable performance of the ChR2 and control animal in the Open Field test with and without phasic dopaminergic activation (Supplementary Figure S2D), suggests that the observed effects in the SCPR test could not have been solely dependent on non-specific changes in the rats’ locomotor output.

A limitation of our study was the use of only one stimulation protocol. It has been previously shown that tonic stimulation of dopaminergic neurons in the VTA results in inhibition of reward consummatory behaviors (Mikhailova et al., 2016) and that tonic but not phasic stimulation reduces ethanol self-administration (Bass et al., 2013). Therefore, it would be interesting to test different stimulation protocols in future studies (e.g., tonic vs. phasic) in relation to their effects on reward-seeking and social competition behaviors.

A remaining question is how the results of this study concerning dominance relationships in dyads could be generalized in the context of the more complex hierarchies and interactions in ethologically relevant (and more naturalistic) settings. Results from such a setting, namely the VBS, in which male and female rats are housed together and social hierarchies are formed rapidly (Blanchard et al., 1995), have suggested that following exposure to the VBS, a subgroup of subordinate males develop alterations in DA activity in mesolimbic structures (Lucas et al., 2004). In addition, dominant males in the VBS display increased responding for food rewards, suggesting higher engagement in reward-seeking behaviors (Davis et al., 2009). Based on this evidence, we could hypothesize that SL rats could possibly assume dominant positions in such naturalistic context, whereas LL rats would most likely be subordinate. However, this needs to be tested experimentally.

In summary, our results provide new insights into the role of reward-seeking behavior in hierarchy establishment. Our study is the first one to highlight that the individual trait seeking behavior, measured by the latency to obtain rewards can be predictive of the outcome of social competition tests after hierarchy has been established. Moreover, our findings suggest a key role for accumbal DA levels in relation to trait reward-seeking behavior after hierarchy establishment. Finally, modulation of dopaminergic neuron firing in the VTA (shown to enhance reward-seeking behaviors), and for the first time, that levels of DA associated to reward acquisition, individually, could determine the outcome of an SCPR test, indicating that reward-seeking behavior and responses to social competition may impinge on the same neuronal circuits.

## Ethics Statement

All experiments were performed with the approval of the Cantonal Veterinary Authorities (Vaud, Switzerland) and carried out in accordance with the European Communities Council Directive of 22 September 2010 (2010/63/EU).

## Author Contributions

CS and LL-M conceived and planned the experiments. IZ, SAb, IGS, and LL-M carried out the experiments. LL-M and CS contributed to the interpretation of the results. LL-M took the lead in writing the manuscript under the supervision of IZ. SAs and IZ provided critical feedback and helped shape the research, analysis and manuscript. All authors gave their inputs to the final version of the manuscript.

## Conflict of Interest Statement

The authors declare that the research was conducted in the absence of any commercial or financial relationships that could be construed as a potential conflict of interest.
