# Supplementary material for: Latency to Reward Predicts Social Dominance in Rats: A Causal Role for the Dopaminergic Mesolimbic System
Source: Front Behav Neurosci. 2019 Apr 5;13:69. doi: 10.3389/fnbeh.2019.00069 (PMC6460316; doi:10.3389/fnbeh.2019.00069)
Supplement: Supplementary file 1 [file Data_Sheet_1.docx]

***Supplementary Material***

# Supplementary Figures and Tables

## Supplementary Figures


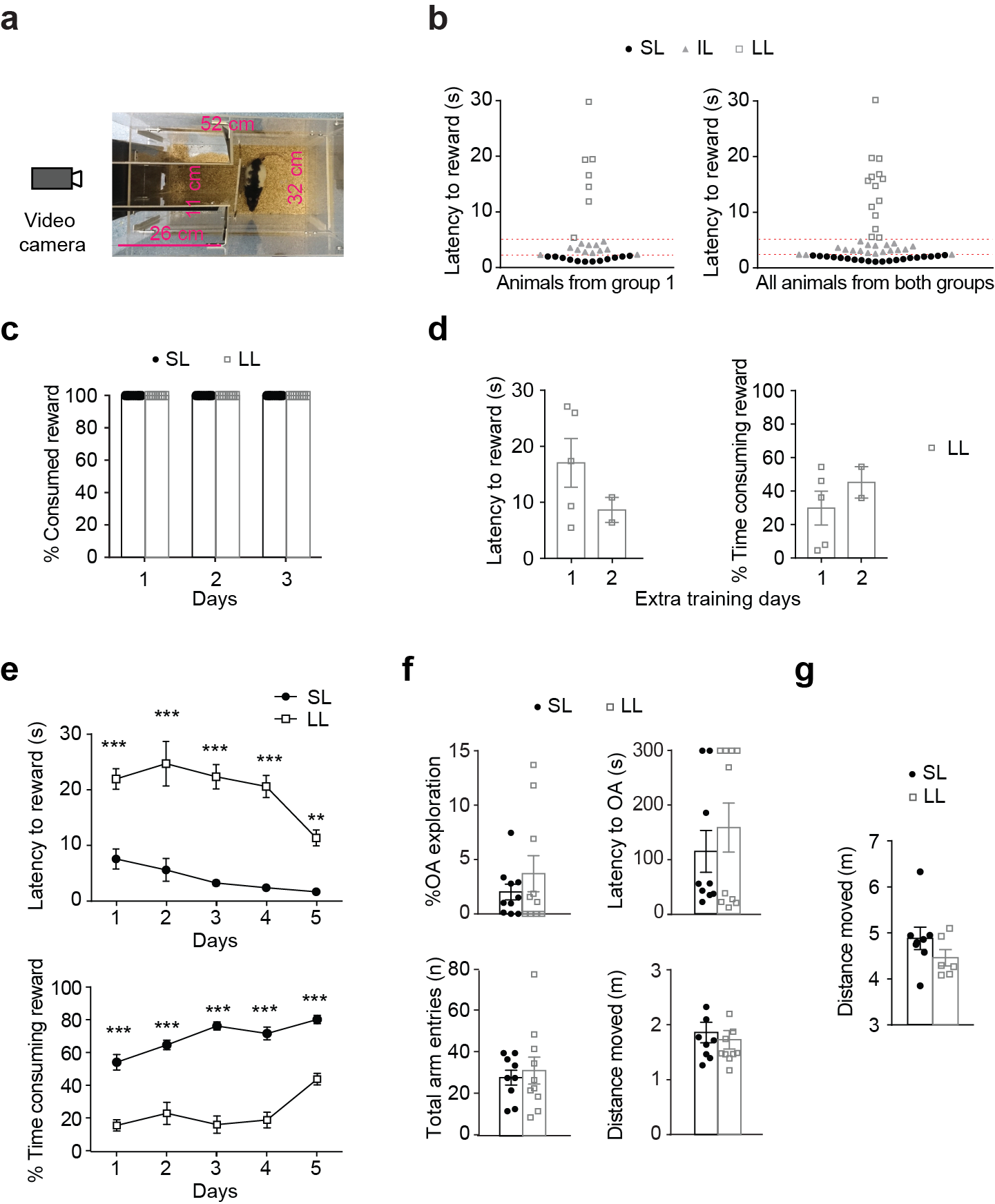


**Supplementary Figure 1.** **Arena description and behavioral measurements from the social competition test. Behavioral analysis of the anxiety and locomotion tests.** (a) Apparatus used for the social competition for palatable rewards test. The runway was sufficiently narrow (11 cm wide) as to allow only one rat at a time to fully enter. At the end of the narrow runway rats could find the drop of chocolate reward. Behavior was recorded through a video camera outside the box on the end of the runway side. (b) The left panel shows the distribution of average latencies during the last day of individual training from a first group animals (n=31). Cut-off values over 75th percentile (5s) were taken for long latency (LL) animals and all animals under the 40th percentile (2.25s) were considered short latency (SL) animals. Intermediate latency (IL) animals (with an average latency over 2.25 s but smaller than 5 s) were not used in our study. The right panel shows the distribution of average latencies during the last day of individual training from the total population of animals used in our study (n=52). Using the cut-off values based on the first group resulted in a very similar distribution. SL animals scored up to the 37th percentile and LL animals scored over the 73th percentile.(c) Chocolate rewards were introduced in the homecages of single housed animals during three consecutive days before the individual training started. All animals consumed the chocolate rewards. (d) Extra training days for animals that did not reach the criterion on day 5 of individual training. Latency to rewards and percentage of time consuming the reward on the two extra days of training for LL animals (each animal is represented as a square in the graph). (e) Average latency to rewards and percentage of time consuming the reward (mean ± SEM) during each of the last 5 days of individual training for SL and LL animals.(f) All animals underwent an EPM test while single housed. Percentage of exploration of open arms, latency to enter to first open arm, total number of arm entries and distance moved on EPM (mean ± SEM) of SL (n=10) and LL (n=10) animals before cohabitation were analyzed. (g) Locomotion measurements of SL and LL animals. A subset of animals underwent an Open Field test prior to SL and LL characterization during the individual training to palatable rewards. Distance moved in the open field test (mean ± SEM) did not differ among SL (n=7) and LL (n=6) animals. ***p*<0.01, ****p*<0.001.


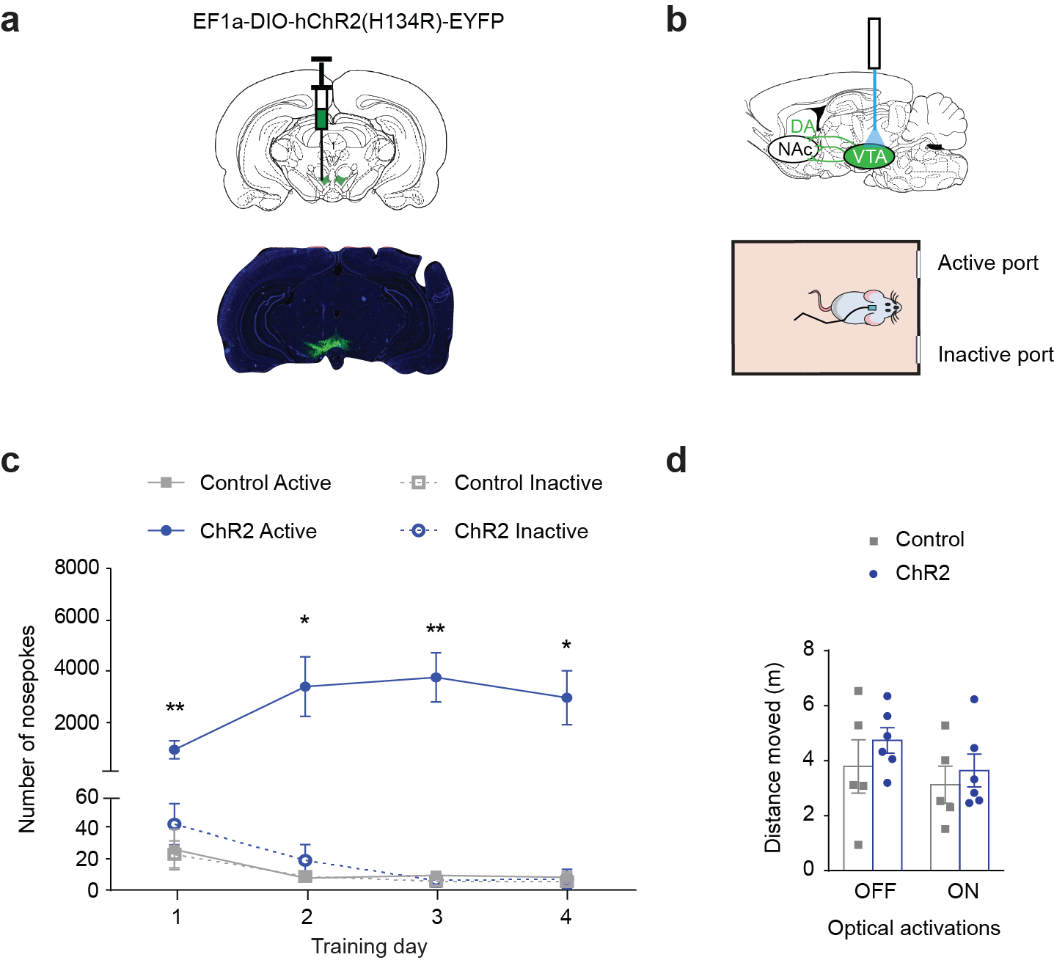


**Supplementary Figure 2. Phasic activation of VTA dopaminergic neurons mediated positive reinforcement by intracranial self-stimulation (ICSS) and do not induce changes in locomotor behaviors on the Open Field test.** (a) Top panel, Cre-dependent adeno-associated virus was injected intra-VTA, unilaterally. Bottom panel, ChR2-eYFP expression in coronal slices shows specific expression within the VTA. (b) Scheme of the operant boxes protocol. (c) Responses into the active port resulted in unilateral phasic stimulation of dopaminergic VTA neurons while responses in the inactive port were without consequence. Number of nosepokes (mean ± SEM) per day of training in ChR2 (n=5) and control (n=5) animals. ChR2 rats performed significantly more nosepokes in the active nosepoke compared to control rats. (d) Effects of phasic activation of VTA dopaminergic neurons on locomotion. Open Filed test was divided in two epochs. The first five minutes were without stimulation (light off), while the second epoch (minutes 5-10) rats underwent optogenetic stimulation (light on). Distance moved in the open field test (mean ± SEM) did not differ among ChR2 (n=6) and Ctrl (n=5) animals regardless of stimulation. *p<0.05, **p<0.01.


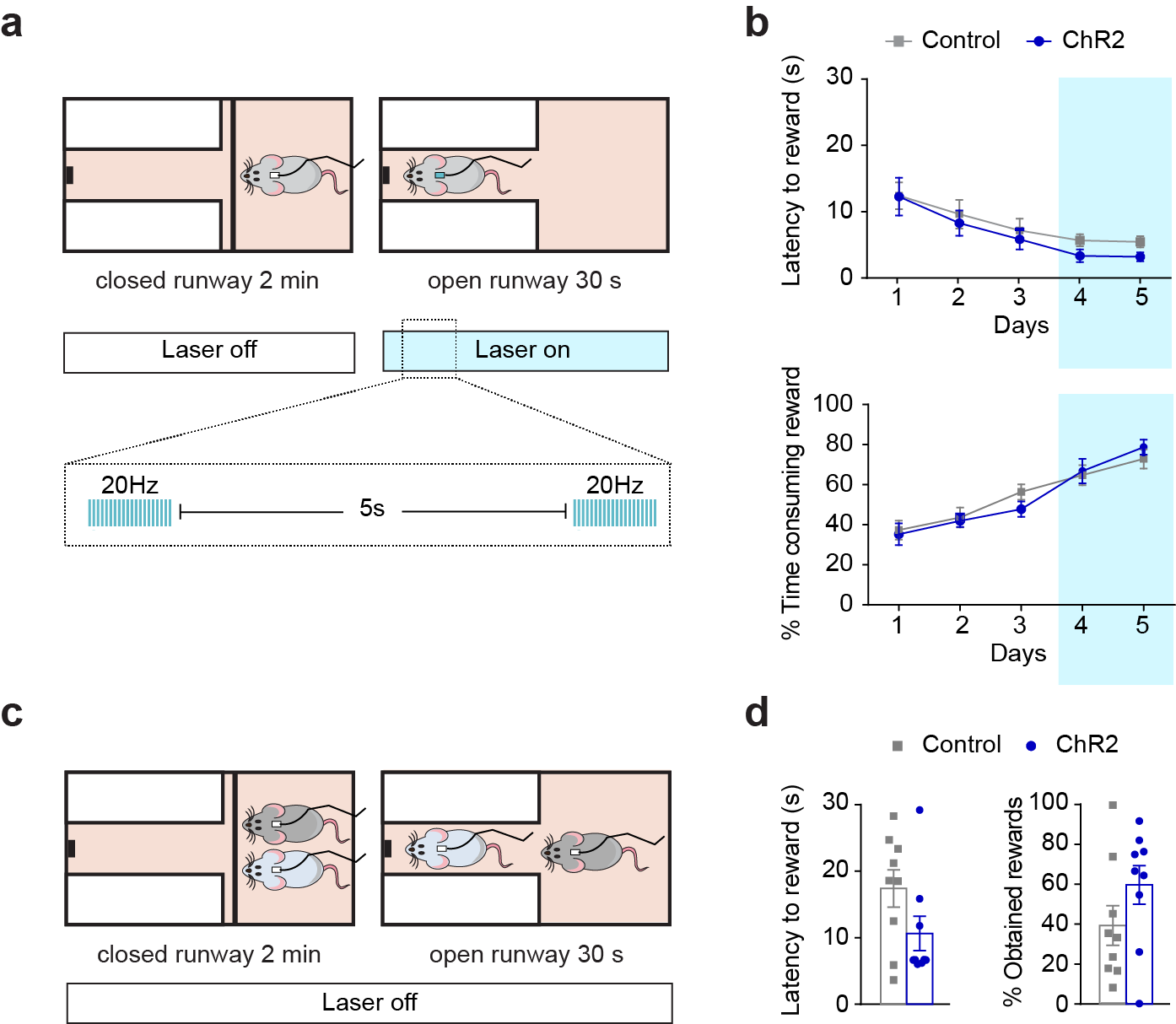


**Supplementary Figure 3. Phasic dopamine activations during individual training had no effect on SCPR test.** (a) In a cohort of rats, ChR2 (n=4) and control (n=4) animals underwent DA activations during the last two days of the individual training, without being further used for a second SCPR test. (b) Average latency to rewards and percentage of time consuming the reward (mean ± SEM) during each day of individual training for all the ChR2 (n=9) and control (n=9) animals from the study (including the animals of Figure 3). Optogenetic stimulation did not result in differences in performance during training. Two way ANOVA followed by Bonferroni post-tests indicated no differences between control and ChR2 animals on their latency to rewards (Group effect: F _(4, 79)_ = 0.11423, p = 0.9772, t= 0.053472, p > 0.99; t= 0.053472, p > 0.99; t= 0.55003, p > 0.99; t= 0.79102, p > 0.99; t= 0.93861, p > 0.99, for days 1-5 respectively); or in the time spent consuming rewards (Group effect F _(4,79)_ = 0.50464, p =0.7324, t= 0.29102, p > 0.99; t= 0.22269, p > 0.99; t= 1.1597, p > 0.99; t= 0.16309, p > 0.99; t= 0.79775, p > 0.99) on any training day. (c) Animals underwent a social competition test without optical activation. (d) No difference in average latency to rewards and percentage of obtained rewards (mean ± SEM) for ChR2 (n=9) and control (n=9) animals during a social competition test. Animals showed equivalent latency to reward (t =1.324, df = 8, p= 0.2220) and percentage of obtained rewards (t = 1.037, df = 8, p = 0.3300). Blue background on the graphs indicates sessions during which optical activation was used. All graphs here also include the animals shown in Figure 3.

## Supplementary Tables


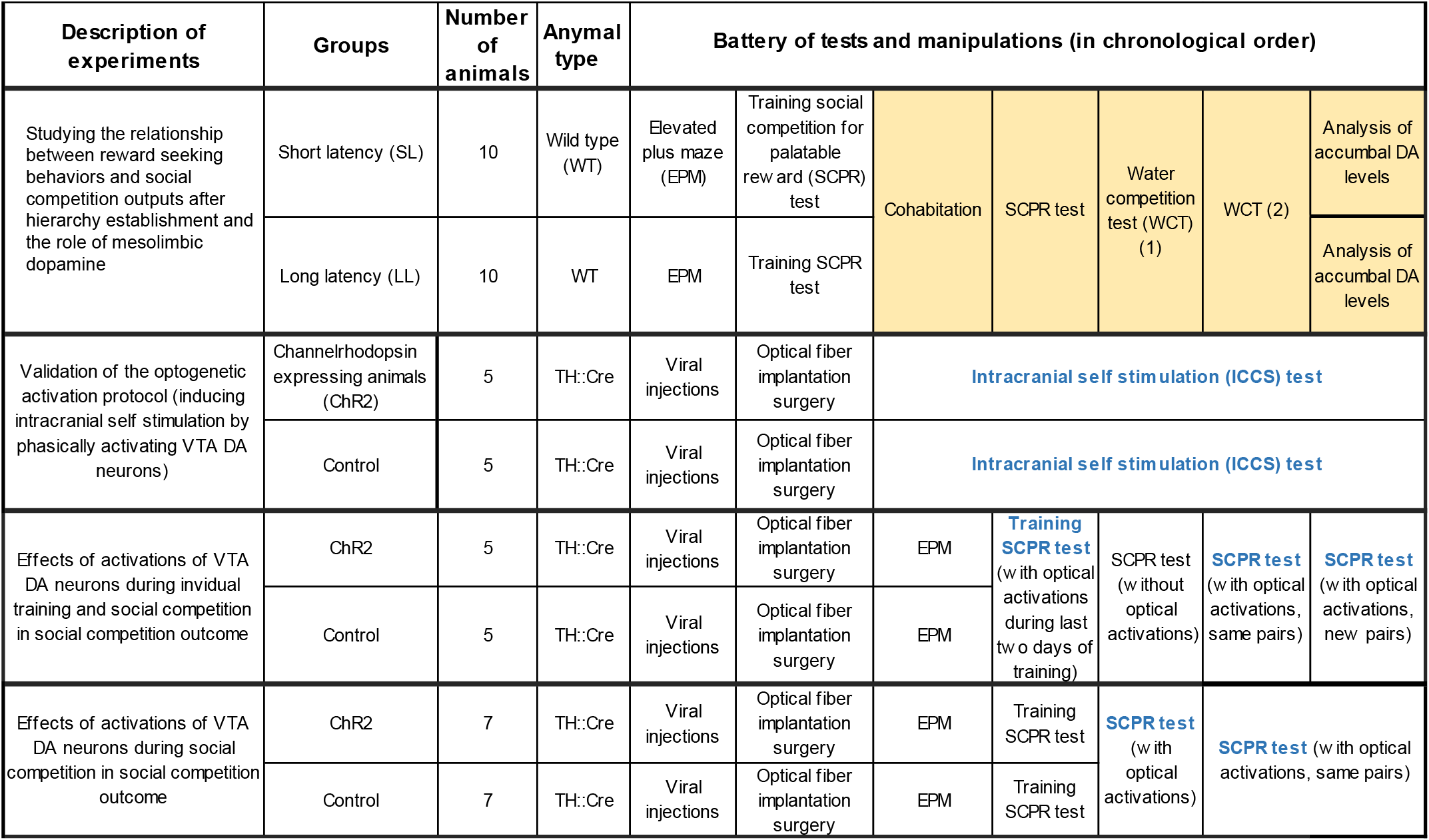


**Supplementary Table 1.** **Overview of different groups of animals used for the experiments and experimental timeline followed by each group.** In the table each highlighted rectangle represents an experimental cluster, and the columns provide description of the experimental aim, the nomenclature used to identify each group of animals, the number of subjects per group and the type of animals. Furthermore, the battery of tests that each group of animals followed is presented in chronological order. Merged rows on the battery of tests indicate that pairs of animals (one from each group) performed the same experiment at a time. Yellow background on the battery of tests indicates that animals were pair-housed (one from each experimental group) during those experiments. Blue font indicate that during those tests animals received optical activations. A more detailed description of the different tests is given in the materials and method section.


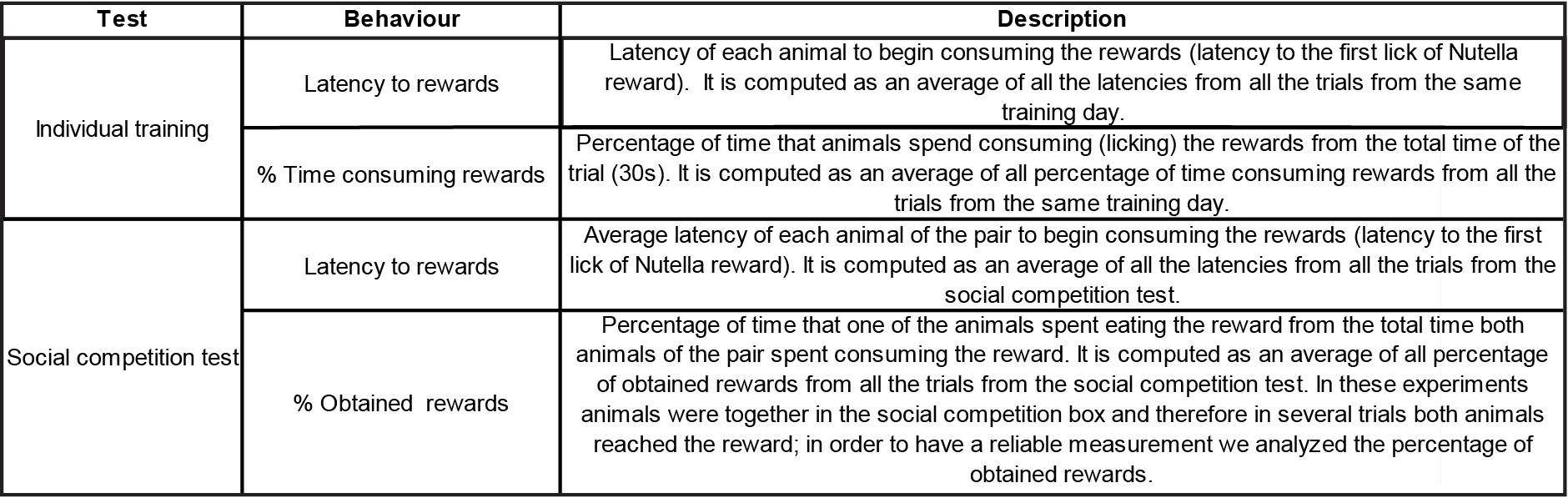


**Supplementary Table 2.** **Overview of scored behaviors for individual training and SCPR test.** In the table each scored behavior for the individual training and SCPR test is described.
